# Polycystic Ovary Syndrome Attenuates TSH-Lowering Effect of Metformin in Young Women with Subclinical Hypothyroidism

**DOI:** 10.3390/ph18081149

**Published:** 2025-08-01

**Authors:** Robert Krysiak, Karolina Kowalcze, Johannes Ott, Sofia Burgio, Simona Zaami, Bogusław Okopień

**Affiliations:** 1 Department of Internal Medicine and Clinical Pharmacology, Medical University of Silesia, Medyków 18, 40-752 Katowice, Poland; 2 Department of Pediatrics in Bytom, Faculty of Health Sciences in Katowice, Medical University of Silesia, Stefana Batorego 15, 41-902 Bytom, Poland; kkowalcze@sum.edu.pl; 3 Department of Pathophysiology, Faculty of Medicine, Academy of Silesia, Rolna 43, 40-555 Katowice, Poland; 4 Clinical Division of Gynecologic Endocrinology and Reproductive Medicine, Department of Obstetrics and Gynecology, Medical University of Vienna, 1090 Vienna, Austria; 5 Maternal and Child Department with Pediatric Emergency Area, Villa Sofia—V. Cervello Hospital, University of Palermo, 90128 Palermo, Italy; 6 Department of Anatomical, Histological, Forensic and Orthopedic Sciences, Sapienza University of Rome, 00161 Rome, Italy

**Keywords:** adenohypophysis, hyperandrogenism, hypothyroidism, insulin sensitivity, metformin, reproductive-aged women

## Abstract

**Background/Objectives:** The effect of metformin on the secretory function of thyrotropic cells is sex-dependent. The current study aimed to investigate whether the impact of this drug on activity of the hypothalamic–pituitary–thyroid axis in women is impacted by the androgen status of patients. **Methods:** The study population included 48 levothyroxine-naïve reproductive-aged women with subclinical hypothyroidism and prediabetes receiving 3.0 g of metformin daily. Women with (*n* = 24) and without (*n* = 24) polycystic ovary syndrome were matched for age, insulin sensitivity, TSH, and reasons for thyroid hypofunction. Circulating levels of glucose, glycated hemoglobin, insulin, TSH, thyroid hormones, gonadotropins, androgens, estradiol, SHBG, prolactin, ACTH, and IGF-1 were measured before metformin treatment and six months later. **Results:** At entry, women with and without polycystic ovary syndrome differed in LH, LH/FSH ratio, androgens, and estradiol. The decrease in TSH, fasting glucose and glycated hemoglobin, and the improvement in insulin sensitivity were less pronounced in women with than in women without polycystic ovary syndrome. In each group, there were no differences in the impact on TSH and thyroid hormones between patients with subclinical hypothyroidism of autoimmune and non-autoimmune origin. The changes in TSH inversely correlated with total testosterone and free androgen index. Only in women with coexisting polycystic ovary syndrome, did metformin slightly reduce LH, LH/FSH ratio, testosterone, and free androgen index. **Conclusions:** The results suggest that concurrent polycystic ovary syndrome attenuates metformin action on TSH secretion, which can be explained by increased androgen production. Moreover, the drug seems to alleviate PCOS-associated changes in the activity of the reproductive axis.

## 1. Introduction

It is well evidenced that metformin decreases the secretory function of overactive anterior pituitary cells, including gonadotropic, lactotropic, and thyrotropic cells [1,2,3,4,5,6,7]. The decrease in thyroid-stimulating hormone (TSH) levels was reported in patients with hypothyroidism of autoimmune and non-autoimmune origin [8]. So far, five factors have been identified to affect metformin action on the hypothalamic–pituitary–thyroid axis. Firstly, the strength of metformin action correlates with baseline secretory activity of thyrotropic cells, and no changes were observed in subjects with TSH levels within the reference range [5,6]. Secondly, this effect seems to be modulated by comorbidities; the decrease in TSH levels was more pronounced if subclinical hypothyroidism was accompanied by prolactin excess [9] and less pronounced in case of coexisting vitamin D deficiency/insufficiency [10]. Thirdly, the impact of metformin on circulating TSH was potentiated by other drugs: statins (rosuvastatin) [11] and dopamine agonists (bromocriptine) [12]. Fourthly, the strength of action probably depends on the sex hormone milieu, mainly on testosterone and estrogen levels, because the effect on secretory function of thyrotropic cells in levothyroxine-naïve subjects with isolated subclinical hypothyroidism was sex-dependent and statistically significant only in women [13]. Lastly, the impact of this drug on TSH levels was found to be dose-dependent [9,10,11].

Polycystic ovary syndrome (PCOS) is considered the most common endocrine disorder, as well as a leading cause of both androgen excess and relative hyperestrogenism in young female population [14,15]. Treatment of PCOS targets the reproductive, cutaneous, metabolic, and psychological complications, and is tailored to individual need [14,15]. However, women with this disorder were also found to be characterized by higher TSH levels than women without PCOS [16]. Moreover, a meta-analysis of six studies, including 692 patients with PCOS and 540 controls, demonstrated that PCOS was strongly associated with an increased risk of subclinical hypothyroidism [17]. Lastly, the prevalence of autoimmune thyroiditis, the major cause of thyroid hypofunction, and the prevalence of patients positive for thyroid antibodies were significantly higher in patients with PCOS than in controls [18]. These findings indicate that many women with PCOS have elevated TSH levels. Interestingly, both subclinical hypothyroidism [19,20] and PCOS [21,22] predispose to type 2 diabetes and metabolic syndrome. Thus, individuals with concurrent subclinical hypothyroidism and PCOS often require chronic treatment with metformin. In addition to beneficial metabolic effects, this drug may be recommended for reproductive-aged women with PCOS in order to stimulate ovulation and to reduce androgen levels [23,24], and may be prescribed to individuals with this disorder in combination with a statin or a dopamine agonist [25,26].

Despite all these theoretical premises, the few studies assessing metformin action on the hypothalamic–pituitary–thyroid axis in women with coexisting thyroid hypofunction and PCOS provided inconsistent results. In the earliest study, metformin reduced TSH concentrations in women with subclinical hypothyroidism and PCOS, but the participants were allowed to receive other treatments [27]. Italian authors reported that a decrease in TSH levels was significant only if metformin was administered in combination with inositol [28]. In another Italian study, metformin decreased circulating levels of TSH, but the study included only nine women with PCOS and thyroid hypofunction, seven of whom were on thyroid replacement therapy [29]. In adolescent girls with this syndrome, metformin decreased elevated levels of TSH, but all participants received additionally spironolactone and pioglitazone [30]. Thus, it is difficult to determine which of these drugs was responsible for lowering TSH. Lastly, TSH levels during pregnancy were unaffected by metformin treatment from the first trimester to delivery, although free thyroxine levels were significantly higher in women randomized to metformin than in women randomized to placebo [31].

A small number of studies, their limitations and inconsistent results, and the likely clinical relevance encouraged us to compare metformin action on the hypothalamic–pituitary–thyroid axis in reproductive-aged levothyroxine-naïve women with classic PCOS and without this syndrome, and to investigate whether pituitary effects of metformin depend on the androgen and estrogen status of patients. High-dose metformin treatment, used in the current study, not only exerted the strongest effect on plasma TSH levels in women with hypothyroidism and normal ovarian function, but also improved glucose tolerance and was well tolerated by patients with prediabetes [9,10,32].

## 2. Results

Two participants (one from each of the study groups) prematurely terminated the study due to adverse effects of metformin. One woman with PCOS was withdrawn because she required chronic treatment with non-steroidal anti-inflammatory drugs. Lastly, another patient (from the control group) dropped out because of changing their place of residence. Consequently, 22 patients in each group (91%) completed the study, and their results were subjected to statistical analysis. Power calculation showed that our sample had 84% power to identify the anticipated difference in the primary outcome. All analyzed patients adhered to the treatment recommendations. Cumulative metformin dose did not differ between the groups (519 ± 11 g vs. 520 ± 10 g, *p* = 0.7539).

There were no between-group differences in age, reasons for hypothyroidism, smoking habits, body mass index, and blood pressure (both systolic and diastolic) (Table 1). Groups I and II differed in luteinizing hormone (LH), LH/follicle-stimulating hormone (FSH) ratio, testosterone, free androgen index (FAI), dehydroepiandrosterone-sulfate (DHEA-S), androstenedione, and estradiol, but not in glucose, glycated hemoglobin (HbA_1c_), the homeostatic model assessment 1 of insulin resistance (HOMA1-IR), TSH, free thyroid hormones, total thyroid hormones, FSH, sex hormone-binding globulin (SHBG), prolactin, adrenocorticotropic hormone (ACTH), and insulin-like growth factor-1 (IGF-1) (Table 2).

In group I, metformin decreased fasting glucose, HbA_1c_, HOMA1-IR, TSH, LH, LH/FSH ratio, testosterone, and FAI, increased SHBG, and had a neutral effect on free and total thyroid hormones, FSH, DHEA-S, androstenedione, estradiol, prolactin, ACTH, and IGF-1. In group II, the drug decreased fasting glucose, HbA_1c_, HOMA1-IR, and TSH, increased SHBG, and did not affect free thyroxine, free triiodothyronine, total thyroxine, total triiodothyronine, LH, FSH, LH/FSH ratio, testosterone, FAI, DHEA-S, androstenedione, estradiol, prolactin, ACTH, and IGF-1. Women with and without PCOS differed in follow-up values of glucose, HbA_1c_, HOMA1-IR, TSH, testosterone, FAI, DHEA-S, androstenedione, and estradiol (Table 2). Neither in group I nor in group II did metformin affect body mass index, systolic blood pressure, and diastolic blood pressure.

There were differences between women with and without PCOS in the percentage changes from baseline in glucose, HbA_1c_, HOMA1-IR, TSH, LH, LH/FSH ratio, testosterone, and FAI (Table 3).

There were no differences in the impact of PCOS on metformin action on plasma glucose, HbA_1c_, HOMA1-IR, TSH, thyroid hormones, LH, LH/FSH ratio, testosterone, and FAI between patients with hypothyroidism of autoimmune and non-autoimmune origin (Table 4).

**Figure 1:**
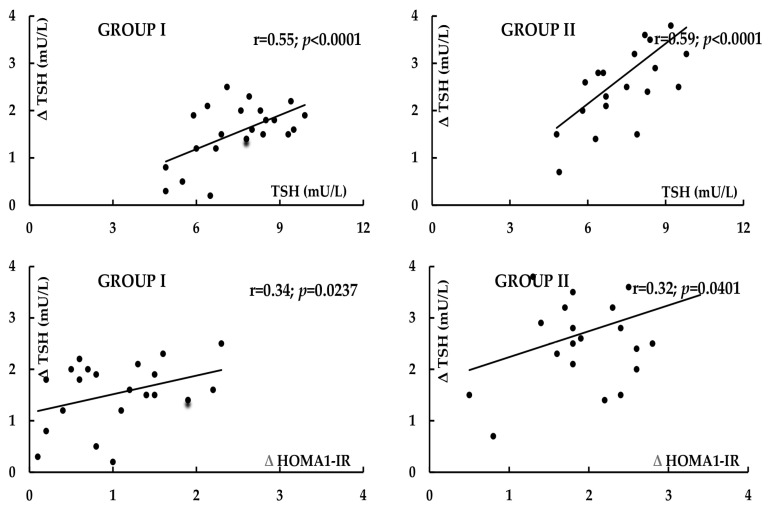
Correlations between the impact of metformin on TSH levels and baseline TSH levels, and between the impact of metformin on TSH levels and on HOMA1-IR. Group I: women with polycystic ovary syndrome. Group II: women without hyperandrogenism, ovulatory dysfunction, and polycystic ovary morphology.

**Figure 2:**
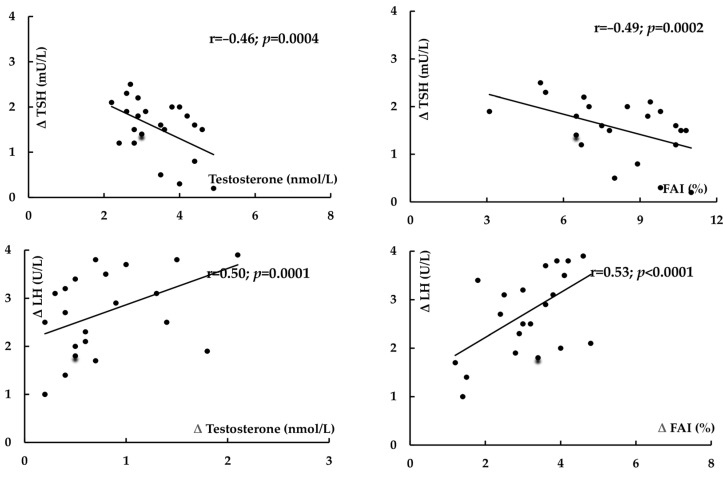
Correlations between the impact of metformin on TSH levels and baseline androgen status, and between the impact of metformin on LH levels and on androgen status in women with subclinical hypothyroidism and polycystic ovary syndrome.

In both study groups, treatment-induced changes in TSH positively correlated with baseline concentrations of this hormone (group I: r = 0.55, *p* < 0.0001; group II: r = 0.59, *p* < 0.0001) and with the impact of treatment on HOMA1-IR (group I: r = 0.34, *p* = 0.0237; group II: r = 0.32, *p* = 0.0401) (Figure 1). In women with PCOS, there were inverse correlations between the decrease in TSH and baseline values of testosterone (r = −0.46, *p* = 0.0004) and baseline FAI (r = −0.49, *p* = 0.0002). In group I, the impact of treatment on LH and LH/FSH ratio positively correlated with the metformin-induced decrease in testosterone and FAI (r = 0.50, *p* = 0.0001 between Δ LH and Δ testosterone; r = 0.53, *p* < 0.0001 between Δ LH and Δ FAI) (Figure 2). In women with PCOS, positive correlations were also observed between the impact on the LH/FSH ratio and the changes in testosterone (r = 0.43, *p* = 0.0008) and in FAI (r = 0.41, *p* = 0.0014).

## 3. Discussion

In line with previous observations [5,6,7,8], metformin decreased TSH concentration in both groups of individuals with subclinical hypothyroidism. Moreover, the drug reduced LH levels and the LH/FSH ratio, which were higher in women with than in women without PCOS. These findings indicate that metformin alleviates overproduction of anterior pituitary hormones. The limitation of metformin action to overactive pituitary cells may also explain why the drug had a neutral effect on plasma levels of FSH, prolactin, and ACTH, which at baseline were within normal limits. Lastly, a neutral effect on IGF-1, a primary mediator of the effects of growth hormone [33], seems to suggest no changes in growth hormone secretion, which is also in line with this explanation. Another observation worth mentioning was the lack of correlations between the impact of metformin on TSH and the effect on LH and the LH/FSH ratio. Thus, it seems that this agent affects different populations of pituitary cells in an independent manner.

Strong positive correlations between the impact on TSH and baseline concentration of this hormone show that the strength of metformin action on TSH depends on the degree of its oversecretion. However, it should be stressed that even in women without hyperandrogenism, ovulatory dysfunction, and polycystic ovarian morphology, treatment-induced changes in TSH were limited, and mean follow-up values of this hormone were still above the upper limit of the reference range. We also observed correlations between metformin-induced changes in TSH and the impact of treatment on insulin sensitivity, which was impaired before the study began. The decrease in TSH levels does not seem to be associated with the concurrent non-pharmacological treatment. All participants complied with the lifestyle modification at least three months before the beginning of the present study, and we did not previously observe changes in hypothalamic–pituitary–thyroid axis activity in metformin-naïve women following dietary and physical activity recommendations [13]. Thus, metformin may be considered an add-on therapy to levothyroxine in case of coexistent disturbances of glucose homeostasis or poor tolerance of high doses of levothyroxine, an alternative to levothyroxine in patients with mild thyroid hypofunction and contraindications to thyroid hormone substitution, and probably also in case of goiter in insulin-resistant women with mild thyroid hypofunction. Although we did not assess changes in the volume of the thyroid gland, the last indication is based on theoretical premises. Both elevated TSH concentrations [34] and impaired insulin sensitivity [35] increase the risk of thyroid enlargement and nodular thyroid hyperplasia, while metformin treatment was found to decrease the prevalence and incidence of goiter [36] and to reduce thyroid nodule size [37].

The inconsistent results of previously conducted studies assessing the impact of metformin on TSH in women with PCOS [27,28,29,30,31] may be explained by important shortcomings in the methodology or study design. Many individuals received levothyroxine substitution and often also other drugs, which might have affected the obtained results. In all studies, the average metformin dose was low or moderate (between 850 and 1500 mg daily) and sometimes differed between patients. Lastly, they included only individuals with PCOS, often with non-classic types (phenotypes C and D), making it almost impossible to determine whether the impact on the hypothalamic–pituitary–thyroid axis was related to PCOS or only reflected baseline activity of this axis in individuals with subclinical hypothyroidism. The above research limitations were eliminated in our study. Thus, the important novel finding of the present study are differences in the strength of metformin action between reproductive-aged women with classic PCOS and control women without hyperandrogenism, ovulatory dysfunction, and polycystic ovarian morphology. Smaller changes in TSH concentration in women with PCOS resemble the impact of this disorder on the prolactin-lowering effect of metformin in young women with prolactin excess [38]. Between-group differences in the TSH-lowering effect of metformin seem to be a consequence of differences in testosterone levels. In line with this explanation, despite a reduction in response to metformin treatment, not only at the beginning but also at the end of the study, testosterone levels and FAI were higher in women with PCOS than in their peers without this disorder. Moreover, elevated values of testosterone and FAI in women with PCOS inversely correlated with the impact of treatment on TSH. Similar relationships were not found for other androgens (androstenedione and DHEA-S) and for estradiol, although circulating levels of these hormones were, throughout the study period, higher in the former group of patients. However, we cannot fully exclude the impact of estrogen produced in peripheral tissues outside the ovaries.

The obtained results allow us to draw some practical conclusions concerning the impact of metformin on the hypothalamic–pituitary–thyroid axis in women with PCOS. Firstly, they suggest that poor TSH response to metformin in women with subclinical thyroid hypofunction may be a marker of concomitant PCOS. Secondly, considering inverse correlations between the decrease in TSH and baseline values of testosterone and FAI, a normal response to metformin may be observed in women with PCOS receiving combination oral contraceptives and/or antiandrogens (spironolactone, cyproterone acetate, flutamide, or finasteride). Thirdly, because even subclinical hypothyroidism is associated with an increased risk of fertility problems and unfavorable pregnancy complications [39], all women with subclinical hypothyroidism and ovulatory dysfunction receiving metformin should be treated simultaneously with adequate doses of levothyroxine. Lastly, coexisting untreated PCOS may prevent other effects of metformin associated with lowering TSH levels (the reduction in goiter incidence, size, and nodularity).

Considering that the presence of PCOS predisposes those with the condition to autoimmune disorders, and one of its features is low-grade inflammation [40], weaker effects of metformin in this group of patients might have theoretically been explained by the impact of thyroid autoimmunity. However, this explanation does not seem convincing. Because of the matching procedure, both study groups did not differ in the percentage of women with autoimmune hypothyroidism. What is more, there were no differences in the impact of PCOS on metformin action on TSH between patients with thyroid hypofunction secondary to Hashimoto’s thyroiditis and of other origin. Lastly, women with and without thyroid autoimmunity did not differ in the impact of metformin on TSH in women without PCOS, in whom baseline levels of this hormone were in the range between 4.5 and 7.5 mU/L [8]. Interestingly, the origin of hypothyroidism in women with concomitant PCOS did not also determine the effect of metformin on LH secretion and on the LH/FSH ratio.

There are some possible explanations for our findings. Both thyrotropic and gonadotropic cells are characterized by the highest expression of adenosine 5′-monophosphate-activated protein kinase (AMPK) among all types of pituitary cells [41]. Thus, it is likely that PCOS alleviates the impact of metformin by interaction at the level of the AMPK pathway in thyrotropic cells. Many arguments indirectly support this explanation. Firstly, owing to the absence of the blood–brain barrier in the pituitary, this brain region accumulates larger amounts of metformin than other brain structures (both after acute and chronic administration) [42,43]. Secondly, the AMPK pathway is an important mediator of metformin action on energy metabolism, which is observed already after therapeutic doses of this drug [44]. Thirdly, AMPK presence in gonadotropic cells was reported to mediate the gonadotropin-lowering effect of metformin in rodents in response to physiological stimulators of FSH and LH secretion [41]. Fourthly, the expression of AMPK is diminished in PCOS, and down-regulation of AMPK signaling pathway predisposes to increased ovarian steroidogenesis, polycystic changes in the ovaries and anovulation/oligoovulation [45]. Lastly, testosterone was reported to down-regulate the AMPK pathway in an animal model of PCOS [46]. The second possible mechanism responsible for a weak TSH-lowering effect of metformin in women with PCOS is associated with the impact on dopamine neurons that project from the tuberal region of the hypothalamus to the median eminence, and with dopamine release at this site that decreases TSH secretion [47]. Metformin administration to women with PCOS was associated with the improvement in dopamine tone in tuberoinfundibular neurons [48]. In turn, the opposite effect, namely the decrease in activity of tuberoinfundibular dopamine neurons was caused by testosterone administration to orchidectomized rodents [49]. Interestingly, both hypotheses explain why the decrease in TSH correlated with the decrease in HOMA1-IR, which is a surrogate marker that estimates insulin sensitivity [50]. Down-regulation of the AMPK pathway impairs insulin sensitivity and promotes hyperglycemia, while the opposite effects are induced by AMPK activators [51]. In turn, stimulation of the central dopaminergic pathways plays a key role in improving insulin sensitivity and glycemia control [52].

Theoretically, the impact on pituitary secretory function may be secondary to the action at the level of the thyroid itself. According to this explanation, the putative improvement in thyroid function would alleviate the need for TSH oversecretion. In line with this interpretation, the metformin-induced decrease in TSH (even in women without PCOS) was not followed by a decrease in thyroid hormones. Moreover, human thyrocytes express the androgen receptor [53], which may partially explain differences between both study groups. Lastly, metformin was found to increase thyroid output in reproductive-aged women with autoimmune subclinical hypothyroidism [8]. While metformin may indeed act in this manner, this does not appear to be the main mode of action on TSH. The same study showed that the impact on thyroid’s secretory capacity was absent if subclinical hypothyroidism was induced by partial thyroidectomy, radioiodine therapy, thyroid hypoplasia/hemiagenesis and dyshormonogenesis. Furthermore, the effect on Jostel’s index, estimating the thyrotropic function of the anterior pituitary lobe, was similar in women with hypothyroidism of autoimmune and non-autoimmune hypothyroidism, which suggests that the negative feedback between the impact on thyroid output and TSH production is counterbalanced by the interactions between thyroid hormones and inflammatory mediators [8]. The current study provides further indirect arguments against this explanation. Metformin did not increase plasma levels of free and total thyroxine and triiodothyronine, and there were no correlations between the impact of metformin on TSH and on thyroid hormones. Lastly, testosterone and FAI in women with PCOS correlated with the decrease in TSH, but not with the impact on thyroid hormones.

Some other conclusions can be drawn from our work. Firstly, concurrent PCOS weakens the metabolic benefits associated with metformin therapy in individuals with prediabetes. In women with this syndrome, metformin may be less efficient in diabetes prevention than in other patients at high diabetes risk. Secondly, the presence of positive correlations between the impact of metformin on LH and the LH/FSH ratio and the changes in testosterone and FAI (estimating levels of physiologically active testosterone [54]) indicates that the reduction in testosterone concentration is, at least partially, secondary to the inhibitory effect of this agent at the level of gonadotropic cells. This explanation is also supported by a neutral effect of the drug on testosterone and FAI in control women. Thirdly, changes in pituitary hormones were more pronounced than the changes in target hormones. Metformin did not affect circulating levels of both total and free thyroid hormones, while its effect on androgens was mild and limited to testosterone. Thus, metformin treatment may bring benefits to individuals with other conditions associated with pituitary hormone oversecretion (pituitary tumors or resistance to anterior pituitary hormones). Lastly, the drug did not affect estradiol concentration, produced in increased amounts in women with PCOS [14,15]. Hence, the drug does not seem to protect against consequences of excess estrogen levels, and their restoration requires other treatments.

These findings should be viewed with some caution due to inherent study limitations. Given the limited participant pool, our results should be interpreted as hypothesis-generating and warranting large-scale validation. Because of the study design, the obtained results could have been influenced by selection and confounding bias. Our findings do not allow us to conclude whether metformin action on the hypothalamic–pituitary–thyroid axis is affected by the remaining phenotypes of PCOS (C and D). Unlike sufficient iodine intake, the study population inhabited the area with inadequate selenium supply [55,56]. Thus, the impact of PCOS on metformin action does not have to be the same in women with inadequate iodine and/or adequate selenium intake. Androgens and estradiol were measured using immunoassays, though the gold standard for quantitation of these hormones are spectrometry-based methods [57]. The study design does not provide a mechanistic explanation for our findings. Lastly, it is difficult to conclude whether PCOS impacts metformin action in women after menopause and women with diabetes, who were excluded from the study.

## 4. Materials and Methods

This prospective matched case–control study was conducted in accordance with the Declaration of Helsinki, and the protocol was approved by the institutional review board. All participants provided written informed consent to participate after having received written and oral information about the study.

### 4.1. Study Population

The participants were recruited among women aged from 20 to 50 years with prediabetes and subclinical hypothyroidism. Only women complying with the lifestyle modification for at least three months were considered eligible for enrollment. Prediabetes was defined as fasting glucose between 100 and 125 mg/dL and/or 2 h post-challenge glucose between 140 and 199 mg/dL. Subclinical hypothyroidism was defined as plasma TSH concentration between 4.5 and 10.0 mIU/L coexisting with free thyroid hormone levels within the reference range (free thyroxine between 10.2 and 21.3 pmol/L and free triiodothyronine concentration between 2.1 and 6.4 pmol/L). No patient considered for enrollment had been treated with levothyroxine or metformin. In order to exclude transient hypothyroidism, TSH and thyroid hormones were measured twice, with four-week intervals between both measurements to exclude transient thyroid hypofunction. In order to calculate baseline levels, both results were averaged.

The study included two groups of women with prediabetes, each with 24 participants. Prospective sample size calculation showed that 21 individuals in each group were required to detect a 20% difference in the primary endpoint (between-group difference in TSH concentration) with 80% power at the 5% level of significance. This calculation was performed using G*Power software version 3.1, being a free tool to compute statistical power analyses. Considering possible dropouts, the sample size exceeded the required number of individuals. Group I consisted of women meeting the following criteria of classic PCOS: clinical and/or biochemical hyperandrogenism, oligo-ovulation/anovulation, with or without polycystic ovarian morphology. Clinical hyperandrogenism was defined as the presence of hirsutism (a modified Ferriman-Gallwey score ≥ 8), acne, or androgenic alopecia. In turn, biochemical hyperandrogenism was defined as plasma testosterone and/or free androgen index (FAI) higher than 2 standard deviations above the mean levels of a normal control population. Amenorrhea was defined as the absence of menstruation for three consecutive months, while oligomenorrhea as an intermenstrual interval longer than 35 days and less than eight menstrual bleedings in the past year. Ultrasound criteria used to define polycystic ovary morphology included the presence of at least 12 ovarian follicles between 2 and 9 mm in diameter in either ovary and/or an ovarian volume greater than 10 mL. Group II, used as a control group, included patients not fulfilling any of diagnostic criteria of PCOS (androgen excess, anovulation/oligoovulation, and polycystic ovary morphology). Owing to a greater number of individuals meeting the criteria for the control group (*n* = 68), only some of them were recruited. The aim of this selection, based on the minimum Euclidean distance rule, was to match both study populations for age, reasons for thyroid hypofunction, HOMA1-IR, and TSH concentration. To mitigate the impact of seasonal variations in the measured parameters, similar number of participants were recruited between March and May (seven in group I and six in group II), between June and August (five in group I and six in group II), between September and November (six in group I and seven in group II), and between December and February (six in group I and five in group II).

We excluded subjects with overt hypothyroidism, diabetes, positive antibodies against thyrotropin receptor, other endocrine or autoimmune disorders, cardiovascular diseases (except for non-pharmacologically treated grade 1 hypertension), liver or kidney failure, malabsorption syndromes, any other serious disorders, premature or early menopause, and liver or kidney failure, as well as any those undergoing pharmacological treatment (including levothyroxine and other thyroid hormone preparations).

### 4.2. Study Design

**Figure 3:**
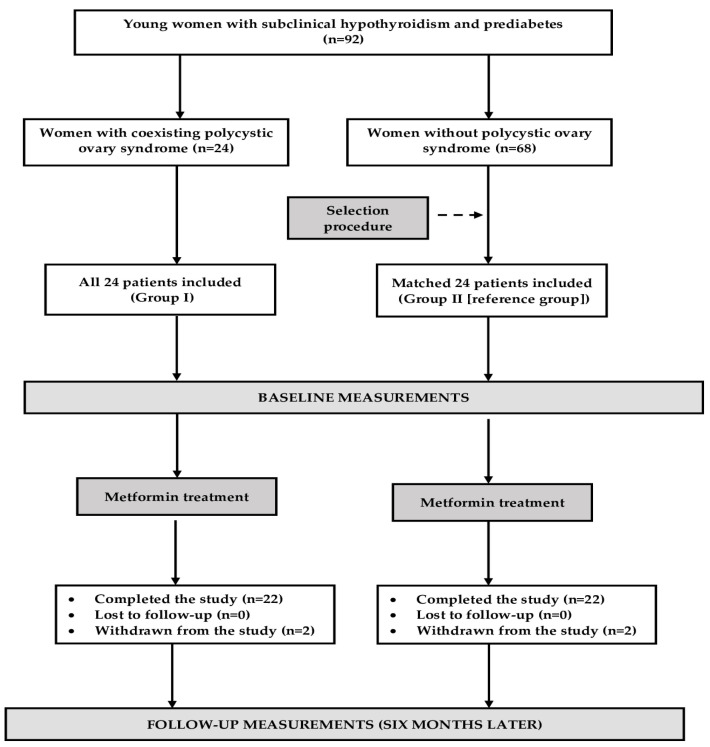
The flow of patients in the study.

Over the entire study period (6 months), both groups of women were treated with metformin and continued to follow the lifestyle modification. Immediate-release tablets of metformin were taken orally with or shortly after meals, without splitting, chewing, or crushing them. The dose of this drug was gradually (every 4–7 days, depending on tolerance) increased from 850 mg once daily to 1000 mg three times a day (3 g daily). This final daily dose, the same in all patients, was administered for the remaining period of time. Adherence to metformin treatment was measured at 8-week intervals by counting the number of tablets returned at the end of each period. The administration of exogenous thyroid hormones was not allowed during the study. Other medications were allowed only if they were used for less than 7 days, and the drug was not administered in the first and last month of the study. Figure 3 shows the flow of patients in the study.

### 4.3. Laboratory Assays

All laboratory assays were conducted at baseline (before metformin treatment) and again six months later. Venous blood samples were obtained from the antecubital vein between 7.30 and 8.30 a.m. after an overnight 12 h fasting. Before venipuncture, the participants had been resting for at least 30 min in the seated position. Plasma was isolated by centrifugation at 1500× *g* for 15 min at 4 °C. All assays were performed in duplicate (to ensure the accuracy and reproducibility of the results) by a technician blinded to the study protocol and the clinical state of the participants, and the results were averaged. Plasma levels of glucose and whole blood content of HbA_1c_ were measured using the multi-analyzer COBAS Integra 400 Plus (Roche Diagnostics, Basel, Switzerland). Plasma levels of insulin, TSH, free, and total thyroid hormones; gonadotropins (LH and FSH); androgens (testosterone, androstenedione and DHEA-S); SHBG; and estradiol were assayed by direct chemiluminescence using acridinium ester technology (ADVIA Centaur XP Immunoassay System, Siemens Healthcare Diagnostics, Munich, Germany). Circulating levels of ACTH and IGF-1 were assayed by solid-phase enzyme-labeled chemiluminescent immunometric assays (Immulite, Siemens, Munich, Germany). The intra-assay and inter-assay coefficients of variations were as follows: 1.5% and 1.9% for glucose, 2.1% and 2.7% for HbA_1c_, 4.9% and 6.3% for insulin, 2.1% and 3.5% for TSH, 2.9% and 4.9% for free thyroxine, 3.2% and 4.8% for free triiodothyronine, 3.2% and 6.1% for total thyroxine, 3.5% and 6.5% for total triiodothyronine, 2.4% and 3.0% for LH, 3.4% and 6.0% for FSH, 4.6% and 5.8% for testosterone, 3.2% and 4.9% for SHBG, 5.0% and 6.2% for DHEA-S, 4.9% and 6.8% for androstenedione, 3.9% and 6.8% for estradiol, 3.0% and 4.9% for prolactin, 3.9% and 8.0% for ACTH, and 4.6% and 5.8% for IGF-1. The assay sensitivities were as follows: 0.6 mg/dL for glucose, 3.9% for HbA_1c_, 0.62 mU/L for insulin, 0.008 mU/L for TSH, 1.3 pmol/L for free thyroxine, 0.3 pmol/L for free triiodothyronine, 3.8 nmol/L for total thyroxine, 0.16 nmol/L for total triiodothyronine, 0.1 U/L for LH, 0.3 U/L for FSH, 0.25 nmol/L for testosterone, 1.7 nmol/L for SHBG, 0.08 μmol/L for DHEA-S, 1.05 nmol/L for androstenedione, 29 pmol/L for estradiol, 0.6 ng/mL for prolactin, 9 pg/mL for ACTH, and 14 ng/mL for IGF-1. HOMA1-IR was calculated by dividing the product of glucose (mmol/L) and insulin (mU/L) and glucose (mmol/L) by 22.5. FAI was calculated by dividing total testosterone (nmol/L) by SHBG (nmol/L) and then multiplying by 100.

### 4.4. Statistical Analysis

Statistical calculations were performed using the Statistica 12.0 PL software (StatSoft Poland, Cracow, Poland). Prior to statistical analysis, all variables were logarithmically transformed to ensure normality. Between-group differences in continuous variables were compared using unpaired *t*-tests, while baseline and follow-up values using paired samples *t*-test. Categorical variables were compared by means of the chi-square test. The relationships between the assessed variables were measured using Pearson’s correlation coefficient. Statistical significance was defined as *p*-value less than 0.05.

## 5. Conclusions

The metformin-induced decrease in TSH concentration and improvement in glucose homeostasis were less pronounced in hypothyroid women with PCOS than in individuals with normal ovarian function. Unlike this finding, the impact on LH, LH/FSH ratio, testosterone and FAI was observed only in women with this disorder. In case of coexistent PCOS, the changes in TSH concentration did not differ between individuals with hypothyroidism of autoimmune and non-autoimmune origin, but inversely correlated with total testosterone and FAI. The obtained results suggest, which is the major novelty and strength of this study, that coexistent PCOS mitigates the impact of metformin on TSH secretion by overactive thyrotropic cells, and possibly also on thyroid’s secretory capacity. The drug may, however, bring hormonal benefits also to women with PCOS, alleviating changes in secretory function of gonadotropic cells and testosterone production associated with this disorder. The obtained results, requiring confirmation in larger longitudinal studies, provide evidence for the interaction between pituitary effects of PCOS and metformin.

## Figures and Tables

**Table 1:** Baseline characteristics of women participating in the study.

Variable	Group I *	Group II **	*p*-Value
Number (*n*)	22	22	-
Age (years)	32 ± 8	34 ± 8	0.4117
Reasons for hypothyroidism (autoimmune/non-autoimmune) (%)	50/50	55/45	0.5014
Smokers (%)/number of cigarettes a day (*n*)/smoking duration (months)	36/9 ± 6/129 ± 41	41/9 ± 7/125 ± 46	0.5238
Body mass index (kg/m^2^)	24.9 ± 4.6	23.9 ± 4.3	0.4605
Systolic blood pressure (mmHg)	127 ± 20	124 ± 19	0.6127
Diastolic blood pressure (mmHg)	84 ± 7	83 ± 6	0.6136

* women with polycystic ovary syndrome. ** women without hyperandrogenism, ovulatory dysfunction and polycystic ovary morphology. Unless otherwise stated, the data are shown as the mean ± standard deviation.

**Table 2:** The impact of metformin on the outcome variables in hypothyroid women with and without polycystic ovary syndrome.

Variable	Group I *	Group II **	*p*-Value
Glucose (mg/dL) [3.89–5.55]			
*Baseline*	6.22 ± 0.34	6.29 ± 0.38	0.5231
*Follow-up*	5.98 ± 0.37	5.72 ± 0.31	**0.0154**
*p-value (follow-up* vs. *baseline)*	**0.0304**	**<0.0001**	-
HOMA1-IR [<2.0]			
*Baseline*	4.1 ± 1.3	3.9 ± 1.4	0.6260
*Follow-up*	3.1 ± 1.2	2.0 ± 1.4	**0.0078**
*p-value (follow-up* vs. *baseline)*	**0.0013**	**0.0001**	-
HbA_1c_ (%) [4.0–5.6]			
*Baseline*	6.0 ± 0.2	6.1 ± 0.2	0.1147
*Follow-up*	5.6 ± 0.2	5.3 ± 0.2	**0.0001**
*p-value (follow-up* vs. *baseline)*	**<0.0001**	**<0.0001**	-
TSH (mU/L) [0.4–4.5]			
*Baseline*	7.5 ± 1.4	7.6 ± 1.3	0.8073
*Follow-up*	6.0 ± 1.5	4.9 ± 1.4	**0.0158**
*p-value (follow-up* vs. *baseline)*	**0.0014**	**<0.0001**	-
Free thyroxine (pmol/L) [10.2–21.3]			
*Baseline*	14.5 ± 2.5	14.2 ± 2.3	0.6808
*Follow-up*	14.9 ± 2.9	15.0 ± 2.8	0.9079
*p-value (follow-up* vs. *baseline)*	0.6267	0.3063	-
Free triiodothyronine (pmol/L) [2.1–6.4]			
*Baseline*	3.4 ± 0.7	3.2 ± 0.7	0.3488
*Follow-up*	3.6 ± 0.8	3.4 ± 0.9	0.5847
*p-value (follow-up* vs. *baseline)*	0.5102	0.5752	-
Total thyroxine (nmol/L) [60–150]			
*Baseline*	98 ± 20	102 ± 25	0.6536
*Follow-up*	106 ± 23	110 ± 30	0.7358
*p-value (follow-up* vs. *baseline)*	0.3594	0.5070	
Total triiodothyronine (nmol/L) [1.2–3.1]			
*Baseline*	1.8 ± 0.3	1.8 ± 0.4	1.0000
*Follow-up*	1.9 ± 0.4	2.0 ± 0.5	0.6156
*p-value (follow-up* vs. *baseline)*	0.4662	0.2664	-
LH (U/L) [2.3–8.4]			
*Baseline*	6.5 ± 2.5	3.1 ± 1.5	**<0.0001**
*Follow-up*	3.7 ± 2.0	3.2 ± 1.7	0.3767
*p-value (follow-up* vs. *baseline)*	**0.0002**	0.8371	-
FSH (U/L) [3.0–9.5]			
*Baseline*	3.7 ± 1.9	3.4 ± 1.4	0.5542
*Follow-up*	3.5 ± 1.5	3.7 ± 1.8	0.6909
*p-value (follow-up* vs. *baseline)*	0.7003	0.5405	-
LH/FSH ratio			
*Baseline*	1.8 ± 0.7	0.9 ± 0.5	**<0.0001**
*Follow-up*	1.1 ± 0.6	0.9 ± 0.4	0.2004
*p-value (follow-up* vs. *baseline)*	**0.0009**	1.0000	-
Testosterone (nmol/L) [0.7–2.4]			
*Baseline*	3.4 ± 0.7	1.3 ± 0.4	**<0.0001**
*Follow-up*	2.6 ± 0.8	1.4 ± 0.4	**<0.0001**
*p-value (follow-up* vs. *baseline)*	**0.0010**	0.4117	-
SHBG (nmol/L) [25–120]			
*Baseline*	42 ± 13	46 ± 12	0.2950
*Follow-up*	52 ± 15	58 ± 16	0.2065
*p-value (follow-up* vs. *baseline)*	**0.0228**	**0.0074**	-
FAI (%) [<5%]			
*Baseline*	8.1 ± 1.5	2.8 ± 1.2	**<0.0001**
*Follow-up*	5.0 ± 2.0	2.4 ± 1.1	**<0.0001**
*p-value (follow-up* vs. *baseline)*	**<0.0001**	0.2556	
DHEA-S (μmol/L) [2.2–10.8]			
*Baseline*	10.5 ± 3.8	5.9 ± 1.9	**<0.0001**
*Follow-up*	9.2 ± 4.1	6.1 ± 2.1	**0.0030**
*p-value (follow-up* vs. *baseline)*	0.2816	0.7421	-
Androstenedione (nmol/L) [1.4–7.8]			
*Baseline*	8.2 ± 2.2	3.8 ± 1.4	**<0.0001**
*Follow-up*	7.3 ± 2.4	3.7 ± 1.5	**<0.0001**
*p-value (follow-up* vs. *baseline)*	0.2019	0.8203	-
Estradiol (pmol/L) [175–640]			
*Baseline*	392 ± 105	280 ± 85	**0.0004**
*Follow-up*	378 ± 112	295 ± 92	**0.0103**
*p-value (follow-up* vs. *baseline)*	0.6710	0.5773	-
Prolactin (ng/mL) [5–28]			
*Baseline*	19 ± 9	15 ± 9	0.1479
*Follow-up*	15 ± 8	13 ± 8	0.4117
*p-value (follow-up* vs. *baseline)*	0.1268	0.4403	-
ACTH (pg/mL) [14–68]			
*Baseline*	36 ± 18	42 ± 14	0.2240
*Follow-up*	40 ± 15	37 ± 16	0.5256
*p-value (follow-up* vs. *baseline)*	0.4278	0.2763	-
IGF-1 (ng/mL) [90–320]			
*Baseline*	220 ± 70	207 ± 73	0.5498
*Follow-up*	200 ± 68	218 ± 64	0.3711
*p-value (follow-up* vs. *baseline)*	0.3419	0.5979	-

* women with polycystic ovary syndrome. ** women without hyperandrogenism, ovulatory dysfunction and polycystic ovary morphology. The data are shown as the mean ± standard deviation. Statistically significant results are marked in bold. Reference values are provided in square brackets.

**Table 3:** Percentage changes from baseline in the outcome variables in hypothyroid women with and without polycystic ovary syndrome.

Variable	Group I *	Group II **	*p*-Value
Δ Glucose	−4 ± 6	−9 ± 8	**0.0238**
Δ HbA_1c_	−9 ± 4	−13 ± 4	**0.0019**
Δ HOMA1-IR	−24 ± 20	−49 ± 31	**0.0028**
Δ TSH	−20 ± 12	−36 ± 18	**0.0012**
Δ Free thyroxine	3 ± 10	6 ± 12	0.3728
Δ Free triiodothyronine	6 ± 9	6 ± 14	1.0000
Δ Total thyroxine	8 ± 11	9 ± 13	0.7843
Δ Total triiodothyronine	6 ± 20	11 ± 24	0.4570
Δ LH	−43 ± 28	3 ± 15	**<0.0001**
Δ FSH	−5 ± 25	9 ± 29	0.0937
Δ LH/FSH ratio	−39 ± 31	0 ± 25	**<0.0001**
Δ Testosterone	−24 ± 26	8 ± 20	**<0.0001**
Δ SHBG	24 ± 20	26 ± 20	0.7418
Δ FAI	−38 ± 29	−14 ± 26	**0.0061**
Δ DHEA-S	−12 ± 32	3 ± 28	0.1055
Δ Androstenedione	−11 ± 29	−3 ± 24	0.3246
Δ Estradiol	−4 ± 28	5 ± 30	0.3175
Δ Prolactin	−21 ± 20	−13 ± 22	0.2139
Δ ACTH	11 ± 28	12 ± 24	0.8994
Δ IGF-1	−9 ± 30	5 ± 29	0.1230

* women with polycystic ovary syndrome. ** women without hyperandrogenism, ovulatory dysfunction and polycystic ovary morphology. The data are shown as the mean ± standard deviation. Statistically significant results are marked in bold.

**Table 4:** Percentage changes from baseline in the selected outcome variables in women with polycystic ovary syndrome and subclinical hypothyroidism of autoimmune or non-autoimmune origin.

Variable	Autoimmune Hypothyroidism	Non-Autoimmune Hypothyroidism	*p*-Value
Δ Glucose	−5 ± 10	−4 ± 8	0.7983
Δ HbA_1c_	−8 ± 2	−8 ± 3	1.0000
Δ HOMA1-IR	−21 ± 23	−27 ± 25	0.5646
Δ TSH	−18 ± 16	−22 ± 15	0.5520
Δ Free thyroxine	4 ± 11	2 ± 13	0.7010
Δ Free triiodothyronine	5 ± 11	7 ± 12	0.6880
Δ Total thyroxine	5 ± 14	11 ± 15	0.3440
Δ Total triiodothyronine	4 ± 18	8 ± 22	0.6457
Δ LH	−39 ± 32	−46 ± 29	0.5968
Δ LH/FSH ratio	−37 ± 40	−41 ± 36	0.8078
Δ Testosterone	−27 ± 21	−21 ± 29	0.5845
Δ FAI	−42 ± 30	−34 ± 34	0.5650

The data are shown as the mean ± standard deviation.

## Data Availability

The original contributions presented in the study are included in the article, further inquiries can be directed to the corresponding author.
